# Assessment of corneal biomechanical properties using Corvis ST following LASIK, PRK, and CXL

**DOI:** 10.1007/s10103-026-04858-9

**Published:** 2026-04-16

**Authors:** Passant Sayed Saif, Mohamed Yasser Sayed Saif, Hisham Mohamed Khairy Abdel Dayem, Mohammed Othman AbdElkhalek Elsayed, Mohamed Abdel Mongey Ibrahim

**Affiliations:** 1 Misr University for Science and Technology, Cairo, Egypt; 2 Beni Suef University, Beni Suef, Egypt; 3 Ain Shams University, Cairo, Egypt; 4 Fayoum University, Al Fayyum, Egypt

**Keywords:** Corvis ST, Corneal biomechanics, LASIK, PRK, Corneal cross-linking, Deformation amplitude, Stiffness parameter, CBI, TBI

## Abstract

To evaluate within-group corneal biomechanical changes induced by LASIK, photorefractive keratectomy (PRK), and corneal cross-linking (CXL) using Corvis ST, while accounting for differences in baseline corneal status between refractive surgery candidates and keratoconic eyes. This prospective comparative study included 150 eyes of 150 patients undergoing LASIK (n = 50), PRK (n = 50), or epithelium-off CXL for keratoconus (n = 50). Corneal biomechanical parameters were assessed preoperatively and at 6 months postoperatively using Corvis ST, including deformation amplitude (DA), stiffness parameter at first applanation (SP-A1), DA ratio at 2 mm, Ambrósio relational thickness horizontal (ARTh), corneal biomechanical index (CBI), and tomographic biomechanical index (TBI), the latter derived from combined Pentacam tomography and Corvis ST measurements. Within-group changes were analyzed using paired parametric or non-parametric tests as appropriate. Between-group comparisons were performed using ANCOVA or linear mixed-effects models adjusted for baseline values, age, central corneal thickness, and biomechanically corrected intraocular pressure. LASIK and PRK eyes demonstrated significant postoperative increases in DA, DA ratio at 2 mm, CBI, and TBI, along with significant reductions in SP-A1 and ARTh (all p < 0.001), indicating biomechanical weakening relative to their preoperative state. The magnitude of weakening was greater following LASIK than PRK after baseline adjustment. In contrast, keratoconic eyes treated with CXL showed significant reductions in DA, DA ratio, CBI, and TBI, and a significant increase in SP-A1 (p < 0.001), consistent with biomechanical stiffening. These changes occurred despite differing baseline biomechanical profiles between groups. LASIK and PRK are associated with measurable corneal biomechanical weakening, whereas CXL induces biomechanical stiffening in keratoconic corneas. These findings should be interpreted within the context of distinct baseline corneal conditions rather than as direct comparisons of procedural efficacy. Corvis ST provides a robust framework for quantifying procedure-specific biomechanical directionality within different clinical settings.

## Introduction

Corneal refractive surgeries and therapeutic interventions have revolutionized the management of refractive errors and ectatic disorders of the cornea. Among these, laser-assisted in situ keratomileusis (LASIK), photorefractive keratectomy (PRK), and corneal cross-linking (CXL) are widely used. Despite their widespread adoption, understanding the biomechanical effects of these procedures on the cornea remains critical, as these changes play a significant role in long-term visual outcomes, refractive stability, and complication rates [1].

Corneal biomechanical properties, including elasticity, viscosity, and stiffness, are essential for maintaining the shape and function under intraocular pressure (IOP). Alterations in these properties may compromise corneal stability, potentially leading to complications such as keratectasia after refractive surgery. The development of devices such as the Corvis ST (Oculus Optikgeräte GmbH) has enabled the precise evaluation of these biomechanical properties. The Corvis ST uses ultra-high-speed Scheimpflug imaging to measure the deformation responses to an air puff.. Commonly used indices derived from Corvis ST include the stiffness parameter at first applanation (SP-A1), the Corvis Biomechanical Index (CBI), the Tomographic Biomechanical Index (TBI), deformation amplitude (DA), DA ratio @2 mm, and Ambrosio’s Relational Thickness horizontal (ARTh). These parameters provide quantitative insight into corneal stiffness and ectasia risk [2, 3].

LASIK is one of the most commonly performed refractive procedures for correcting myopia, hyperopia, and astigmatism. Surgery involves the creation of a corneal flap, followed by stromal ablation using an excimer laser. Although LASIK provides excellent visual outcomes, it alters the corneal architecture and weakens the biomechanical strength by removing the stromal tissue. Studies have shown a significant reduction in DA and central corneal thickness (CCT) postoperatively, which correlates with decreased stiffness and an increased risk of ectasia [4, 5].

PRK is a surface ablation technique that avoids flap creation and removes the corneal epithelium to expose the stroma for laser ablation. While PRK preserves more biomechanical integrity than LASIK, it still causes a measurable reduction in corneal stiffness and thickness. Comparative studies have reported that PRK results in less biomechanical weakening than LASIK, as reflected by smaller changes in DA and higher stiffness parameters [4, 6]. CXL is a therapeutic approach primarily used for keratoconus and other ectatic conditions. This procedure enhances the biomechanical strength of the cornea by inducing collagen cross-linking with riboflavin and ultraviolet-A exposure. Corvis ST–based studies have consistently demonstrated improvements in parameters such as stiffness parameter at the first applanation (SP-A1), corneal biomechanical index (CBI), and tomographic biomechanical index (TBI) following CXL [2, 7].

Although LASIK, PRK, and CXL serve distinct purposes of refractive correction and structural stabilization, they significantly impact corneal biomechanics. Direct comparisons are critical for understanding the scope of these changes and optimizing procedural selection. Corvis ST offers clinicians a valuable platform for quantifying biomechanical alterations across surgical modalities. Recent advances have also examined the benefits of adjunctive CXL with refractive procedures, such as LASIK Xtra and PRK Xtra, which enhance the biomechanical outcomes [6, 8].

Unlike prior studies that primarily report unadjusted postoperative biomechanical changes, this study introduces baseline-adjusted, depth-normalized analyses and clinically oriented risk stratification to quantify the *biomechanical cost* of refractive and therapeutic corneal procedures. This approach shifts the focus from descriptive confirmation to mechanism-based and decision-relevant interpretation.

### Aim of the work

The aim of this study was to quantify within-group corneal biomechanical changes measured by Corvis ST following LASIK, PRK, and corneal cross-linking, while explicitly accounting for baseline differences between healthy refractive surgery candidates and keratoconic eyes. Secondary analyses explored adjusted inter-group differences with careful interpretation of their clinical limitations.

#### Subjects and methods

This prospective comparative study aimed to evaluate and compare corneal biomechanical changes in patients undergoing LASIK, PRK, and corneal cross-linking (CXL). The study was conducted from January 2024 to May 2025.

Eligible participants were adults aged 18–40 years. Patients considered for LASIK or PRK had to be suitable candidates for refractive error correction, whereas those undergoing CXL were required to have a confirmed diagnosis of keratoconus. Additional inclusion criteria included the absence of any prior ocular surgeries and willingness to participate in the study with signed informed consent.

The exclusion criteria comprised the presence of corneal scars, active ocular inflammation, or other corneal pathologies that could interfere with the procedure or outcomes. Patients with systemic conditions, such as diabetes mellitus or connective tissue disorders, were excluded to eliminate potential confounding factors. A history of contact lens wear within the two weeks prior to enrollment was also a basis for exclusion, as was any anticipated inability to complete the follow-up visits or adhere to the study protocol.

All participants underwent comprehensive preoperative ophthalmic evaluation. This included uncorrected and best-corrected visual acuity (UCVA and BCVA) assessments, detailed anterior and posterior segment examinations using slit-lamp biomicroscopy, fundus evaluation after pupillary dilation, and corneal topography to confirm keratoconus or to evaluate refractive errors.

The surgical interventions varied between the groups. In the LASIK group, a corneal flap was created using a femtosecond laser, followed by excimer laser ablation of the stromal bed to achieve the desired refractive correction. In the PRK group, the corneal epithelium was removed, excimer laser ablation was performed on the stromal surface, and a bandage contact lens was applied postoperatively. For patients undergoing CXL, the standard epithelium-off (epi-off) protocol was employed, involving riboflavin instillation followed by ultraviolet-A irradiation at an intensity of 3 mW/cm^2^ for 30 min.

We recorded adjunctive cross-linking performed at the time of refractive surgery (e.g., LASIK-Xtra, PRK-Xtra) and handled these cases via prespecified sensitivity analyses (excluded from primary analyses) because addition of CXL alters immediate biomechanical responses.

Postoperative follow-up was scheduled at 1 week, 1 month, 3 months, and 6 months. Corneal biomechanical assessments were conducted using the Corvis ST device before surgery and at the 6-month postoperative visit for all participants across the three groups.

Corneal biomechanical assessments were conducted using the Corvis ST device (Oculus Optikgeräte GmbH control unit Version 2.01.2281, Measure head version 2.01.2127 Software Version 1.22r05)both preoperatively and at 6 months postoperatively. The measurements included intraocular pressure (IOP)-related parameters, such as Goldmann-correlated IOP (IOPg) and biomechanically corrected IOP (bIOP). The central corneal thickness (CCT) was recorded to evaluate structural changes. The dynamic corneal response parameters included the time to first and second applanation (A1T and A2T), velocity of the corneal surface at first and second applanation (A1V and A2V), deformation amplitude (DA), and radius of curvature at the point of highest concavity (R). In addition, key biomechanical indices were analyzed, including the stiffness parameter at first applanation (SP-A1), corneal biomechanical index (CBI), tomographic biomechanical index (TBI),*DA ratio @2 mm* (ratio comparing central DA to peripheral DA at 2 mm from the apex, indicating non-uniform deformation), and Ambrosio’s Relational Thickness horizontal *(ARTh)*, a pachymetric progression metric reflecting thinning patterns and structural integrity.

Data analysis was performed using IBM SPSS Statistics (version 25). Within-group changes were assessed using paired t-tests or Wilcoxon signed-rank tests, as appropriate. To compare groups while adjusting for baseline differences, we used analysis of covariance (ANCOVA) with the postoperative value as the dependent variable, procedure group as the fixed factor, and the corresponding baseline value, age, baseline CCT, and baseline bIOP as covariates. When ANCOVA assumptions were violated, linear mixed-effects models (subjects as random effects) with the same covariates were used. Sensitivity analyses excluded eyes undergoing adjunctive cross-linking (“Xtra”) and involved stratified analyses based on the baseline ectasia status. Multiple comparisons were corrected using Bonferroni adjustments; p < 0.05 was considered significant.

To ensure transparency and reproducibility, statistical analyses were conducted in accordance with recommended reporting standards for biomedical research. Assumptions for parametric testing were assessed using the Shapiro–Wilk test for normality and Levene’s test for homogeneity of variances. Selection of paired t-tests or Wilcoxon signed-rank tests was based on these diagnostics. ANCOVA models were evaluated for linearity, homogeneity of regression slopes, and absence of multicollinearity. When these assumptions were violated, linear mixed-effects models with subject-level random effects were applied. Multiple comparisons were adjusted using Bonferroni correction, and all effect estimates are reported with corresponding 95% confidence intervals.

This study was conducted in compliance with the ethical standards outlined in the Declaration of Helsinki. Ethical approval was obtained from the Faculty of Medicine Research Ethical Committee (Approval No. FMBSUREC/05012025/SAIF), *and clinical trial registration: ClinicalTrials.gov Identifier:: NCT06818461. Registration date: April 2024.”*. Written informed consent was obtained from all participants before their inclusion in the study. Participants were informed of their right to withdraw from the study at any time without consequences. All patient data were anonymized to ensure confidentiality and data protection.

## Results

A total of 150 eyes from 150 patients were included in this study and divided equally into three groups: LASIK (n = 50), PRK (n = 50), and CXL (n = 50). As summarized in Table 1, there were no statistically significant differences among the three groups in terms of age, sex distribution, or preoperative central corneal thickness (CCT), with p-values greater than 0.05, indicating a well-balanced study population.

**Table 1:** Baseline demographic and clinical characteristics of patients undergoing LASIK, PRK, or CXL

Parameter	LASIK (n = 50)	PRK (n = 50)	CXL (n = 50)	*p*-value
Age (years, mean ± SD)	28.5 ± 5.3	29.2 ± 5.1	27.8 ± 4.8	0.432
Sex, n (%)	Male 28 (56%) Female 22 (44%)	Male 26 (52%) Female 24 (48%)	Male 30 (60%) Female 20 (40%)	0.562
Spherical Equivalent (D, mean ± SD)	− 4.25 ± 1.8	− 4.10 ± 1.9	− 2.10 ± 1.5	< 0.001*
Sphere (D)	− 3.75 ± 2.1	− 3.60 ± 2.0	− 1.90 ± 1.4	< 0.001*
Cylinder (D)	− 1.25 ± 0.7	− 1.35 ± 0.8	− 2.30 ± 1.2	< 0.001*
Preoperative CCT (µm, mean ± SD)	520.3 ± 15.2	518.5 ± 16.7	468.9 ± 12.5	< 0.001*
Mean K (D)	43.2 ± 1.6	43.5 ± 1.7	46.8 ± 2.0	< 0.001*
Steep K (D)	44.1 ± 1.7	44.4 ± 1.6	48.2 ± 2.2	< 0.001*
Flat K (D)	42.4 ± 1.5	42.7 ± 1.6	45.3 ± 1.9	< 0.001*
Preoperative IOP (mmHg, mean ± SD)	15.3 ± 2.1	15.6 ± 2.4	14.9 ± 2.2	0.314
bIOP (mmHg)	16.0 ± 2.2	16.0 ± 1.7	16.2 ± 1.5	0.751

Values are mean ± standard deviation (SD) or n (%)

*CCT* central corneal thickness; *K* keratometry; *IOP* intraocular pressure; *bIOP* biomechanically corrected IOP

*p*-values calculated using ANOVA (continuous) or chi-square (categorical)

^*^p < 0.05 considered statistically significant

As expected, keratoconus eyes (CXL group) had significantly thinner corneas, steeper keratometry, and higher astigmatism compared with refractive surgery candidates (LASIK/PRK)

Both Goldmann-correlated IOP (IOPg) and biomechanically corrected IOP (bIOP) were analyzed preoperatively and postoperatively (Table 2). In the LASIK group, IOPg decreased from 15.3 ± 2.1 mmHg to 13.2 ± 1.5 mmHg (*p* < 0.001), and bIOP decreased from 16.0 ± 2.2 mmHg to 14.7 ± 2.1 mmHg, with a mean difference of 1.28 ± 1.76 mmHg (95% CI, 0.78–1.78; *p* < 0.001). Similarly, in the PRK group, IOPg decreased from 15.6 ± 2.4 mmHg to 13.5 ± 1.4 mmHg (*p* < 0.001), and bIOP decreased from 16.0 ± 1.7 mmHg to 14.7 ± 1.6 mmHg, with a mean difference of **1**.32 ± 1.72 mmHg (95% CI, 0.83–1.81; p < 0.001). In contrast, the CXL group showed no significant change: IOPg remained stable (14.9 ± 2.2 vs. 14.8 ± 1.8 mmHg; *p* = 0.184), and bIOP also did not differ significantly (16.2 ± 1.5 vs. 16.1 ± 3.0 mmHg; mean difference 0.10 ± 2.91 mmHg; 95% CI, − 0.73 to 0.93; *p* = 0.805).

**Table 2:** Comparison of preoperative and postoperative intraocular pressure measured by Goldmann-correlated IOP (IOPg) and biomechanically corrected IOP (bIOP) in LASIK, PRK, and CXL groups Preoperative, postoperative, and ANCOVA-adjusted intraocular pressure (IOPg and bIOP) in LASIK, PRK, and CXL groups

Group	Parameter	Pre-op Mean ± SD (mmHg)	Post-op Mean ± SD (mmHg)	Absolute Change (Δ) Mean ± SD	95% CI of Change	p-value (within group)	ANCOVA Adjusted Comparison (vs other groups)*
LASIK	IOPg	15.3 ± 2.1	13.2 ± 1.5	− 2.10 ± 1.45	(− 2.57 to − 1.63)	< 0.001*	vs PRK: − 0.05 (p = 0.86) vs CXL: − 1.95 (p < 0.001*)
	bIOP	16.0 ± 2.2	14.7 ± 2.1	− 1.28 ± 1.76	(− 1.78 to − 0.78)	< 0.001*	vs PRK: − 0.04 (p = 0.88) vs CXL: − 1.55 (p < 0.001*)
PRK	IOPg	15.6 ± 2.4	13.5 ± 1.4	− 2.05 ± 1.56	(− 2.53 to − 1.57)	< 0.001*	vs LASIK: + 0.05 (p = 0.86) vs CXL: − 1.90 (p < 0.001*)
	bIOP	16.0 ± 1.7	14.7 ± 1.6	− 1.32 ± 1.72	(− 1.81 to − 0.83)	< 0.001*	vs LASIK: + 0.04 (p = 0.88) vs CXL: − 1.51 (p < 0.001*)
CXL	IOPg	14.9 ± 2.2	14.8 ± 1.8	− 0.12 ± 1.12	(− 0.58 to 0.34)	0.184	vs LASIK: + 1.95 (p < 0.001*) vs PRK: + 1.90 (p < 0.001*)
	bIOP	16.2 ± 1.5	16.1 ± 3.0	− 0.10 ± 2.91	(− 0.93 to 0.73)	0.805	vs LASIK: + 1.55 (p < 0.001*) vs PRK: + 1.51 (p < 0.001*)

Values are mean ± standard deviation (SD)

Δ = postoperative – preoperative. Negative values indicate a decrease

*CI* confidence interval

^*^p < 0.05 considered statistically significant

^*^ANCOVA model adjusted for baseline value, age, and central corneal thickness (CCT)

Significant reductions in Central Corneal Thickness (CCT) were observed in the LASIK and PRK groups (*p* < 0.001), consistent with the expected stromal tissue removal (Table 3). The CXL group experienced only a mild decrease in CCT postoperatively (*p* = 0.043), which was attributable to stromal compaction following cross-linking.

**Table 3:** Preoperative, postoperative, and ANCOVA-adjusted central corneal thickness (CCT) in LASIK, PRK, and CXL groups

Group	N	Pre-op CCT (µm)	Post-op CCT (µm)	Absolute Change (Δ) Mean ± SD	95% CI of Change	p-value (within group)	ANCOVA Adjusted Post-op Difference vs Other Groups*
LASIK	50	520.3 ± 15.2	465.8 ± 12.3	− 54.5 ± 10.6	(− 58.0 to − 51.0)	< 0.001*	vs PRK: − 3.2 (p = 0.21) vs CXL: − 34.9 (p < 0.001*)
PRK	50	518.5 ± 16.7	469.2 ± 13.6	− 49.3 ± 11.1	(− 53.0 to − 45.6)	< 0.001*	vs LASIK: + 3.2 (p = 0.21) vs CXL: − 31.7 (p < 0.001*)
CXL	50	468.9 ± 12.5	460.1 ± 10.7	− 8.8 ± 7.4	(− 11.2 to − 6.4)	0.043*	vs LASIK: + 34.9 (p < 0.001*) vs PRK: + 31.7 (p < 0.001*)

*CCT* central corneal thickness

Values are mean ± SD

Δ = postoperative – preoperative. Negative values indicate corneal thinning

*CI* confidence interval

^*^p < 0.05 considered statistically significant

^*^ANCOVA adjusted for baseline CCT, age, and bIOP

Dynamic Biomechanical Parameters: Key corneal biomechanical parameters were evaluated before and after surgery using Corvis ST, as presented in Table 4. The deformation amplitude (DA) significantly increased in the LASIK and PRK groups (*p* < 0.001), indicating reduced biomechanical stiffness. In contrast, the CXL group showed a significant decrease in DA (*p* < 0.001), reflecting increased stiffness. Similarly, the stiffness parameter at first applanation (SP-A1) decreased significantly in the LASIK and PRK groups (*p* < 0.001), whereas it increased significantly in the CXL group (*p* < 0.001), further supporting the notion of reinforcement of the biomechanical properties of the cornea.

**Table 4:** Dynamic biomechanical parameters before and after LASIK, PRK, and CXL, with ANCOVA-adjusted inter-group comparisons

Parameter	Group	Pre-op Mean ± SD	Post-op Mean ± SD	Absolute Change (Δ) Mean ± SD	p-value (within group)	ANCOVA Adjusted Post-op Difference vs Other Groups*
DA (mm)	LASIK (n = 50)	1.14 ± 0.13	1.26 ± 0.15	+ 0.12 ± 0.08	< 0.001*	vs PRK: + 0.05 (*p* = 0.07) vs CXL: + 0.37 (*p* < 0.001*)
	PRK (n = 50)	1.12 ± 0.12	1.21 ± 0.14	+ 0.09 ± 0.07	< 0.001*	vs LASIK: − 0.05 (*p* = 0.07) vs CXL: + 0.32 (*p* < 0.001*)
	CXL (n = 50)	0.92 ± 0.11	0.89 ± 0.12	− 0.03 ± 0.07	0.118	vs LASIK: − 0.37 (*p* < 0.001*) vs PRK: − 0.32 (*p* < 0.001*)
SP-A1 (N/mm)	LASIK (n = 50)	68.7 ± 5.4	63.4 ± 5.1	− 5.3 ± 3.2	< 0.001*	vs PRK: − 1.7 (*p* = 0.08) vs CXL: − 12.5 (*p* < 0.001*)
	PRK (n = 50)	67.0 ± 5.0	65.1 ± 4.8	− 1.9 ± 2.9	0.012*	vs LASIK: + 1.7 (*p* = 0.08) vs CXL: − 10.8 (*p* < 0.001*)
	CXL (n = 50)	64.2 ± 5.5	75.9 ± 6.2	+ 11.7 ± 4.1	< 0.001*	vs LASIK: + 12.5 (*p* < 0.001*) vs PRK: + 10.8 (*p* < 0.001*)
Radius (R, mm)	LASIK (n = 50)	8.4 ± 0.3	8.9 ± 0.4	+ 0.5 ± 0.3	< 0.001*	vs PRK: + 0.3 (*p* = 0.09) vs CXL: + 2.1 (*p* < 0.001*)
	PRK (n = 50)	8.3 ± 0.3	8.6 ± 0.3	+ 0.3 ± 0.2	< 0.001*	vs LASIK: − 0.3 (*p* = 0.09) vs CXL: + 1.8 (*p* < 0.001*)
	CXL (n = 50)	7.0 ± 0.5	6.8 ± 0.5	− 0.2 ± 0.3	0.041*	vs LASIK: − 2.1 (*p* < 0.001*) vs PRK: − 1.8 (*p* < 0.001*)

*DA* deformation amplitude; *SP-A1* stiffness parameter at first applanation; *R* radius of curvature

Values are mean ± SD

Δ = postoperative – preoperative

*CI* not shown for brevity; available on request

^*^p < 0.05 considered statistically significant

^*^ANCOVA adjusted for baseline values, age, and central corneal thickness (CCT)

Biomechanical Indices (CBI and TBI): As shown in Table 5, the LASIK and PRK groups demonstrated significant increases in both the corneal biomechanical index (CBI) and the tomographic biomechanical index (TBI) postoperatively (*p* < 0.001), suggesting biomechanical weakening. Conversely, the CXL group exhibited significant reductions in both indices (*p* < 0.001), indicating enhanced biomechanical integrity.

**Table 5:** Preoperative, postoperative, and ANCOVA-adjusted corneal biomechanical indices (CBI and TBI) in LASIK, PRK, and CXL groups

Parameter	Group	Pre-op Mean ± SD	Post-op Mean ± SD	Absolute Change (Δ) Mean ± SD	95% CI of Change	p-value (within group)	ANCOVA Adjusted Post-op Difference vs Other Groups*
CBI	LASIK (n = 50)	0.33 ± 0.08	0.51 ± 0.12	+ 0.18 ± 0.09	(0.15 to 0.21)	< 0.001*	vs PRK: − 0.02 (*p* = 0.22) vs CXL: + 0.30 (*p* < 0.001*)
	PRK (n = 50)	0.35 ± 0.09	0.55 ± 0.13	+ 0.20 ± 0.11	(0.16 to 0.24)	< 0.001*	vs LASIK: + 0.02 (*p* = 0.22) vs CXL: + 0.32 (*p* < 0.001*)
	CXL (n = 50)	0.45 ± 0.11	0.21 ± 0.09	− 0.24 ± 0.12	(− 0.28 to − 0.20)	< 0.001*	vs LASIK: − 0.30 (*p* < 0.001*) vs PRK: − 0.32 (*p* < 0.001*)
TBI	LASIK (n = 50)	0.49 ± 0.10	0.67 ± 0.15	+ 0.18 ± 0.11	(0.14 to 0.22)	< 0.001*	vs PRK: − 0.02 (*p* = 0.18) vs CXL: + 0.35 (*p* < 0.001*)
	PRK (n = 50)	0.50 ± 0.12	0.69 ± 0.14	+ 0.19 ± 0.12	(0.15 to 0.23)	< 0.001*	vs LASIK: + 0.02 (*p* = 0.18) vs CXL: + 0.37 (*p* < 0.001*)
	CXL (n = 50)	0.61 ± 0.13	0.32 ± 0.11	− 0.29 ± 0.13	(− 0.33 to − 0.25)	< 0.001*	vs LASIK: − 0.35 (*p* < 0.001*) vs PRK: − 0.37 (*p* < 0.001*)

*CBI* Corvis Biomechanical Index; *TBI* Tomographic Biomechanical Index

Values are mean ± SD

Δ = postoperative – preoperative. Positive values = increase, negative values = decrease

*CI* confidence interval

^*^p < 0.05 considered statistically significant

^*^ANCOVA adjusted for baseline values, age, and central corneal thickness (CCT)

Overall, LASIK and PRK procedures were associated with a marked reduction in corneal stiffness, as evidenced by increased DA and decreased SP-A1 levels. In contrast, CXL led to significant biomechanical strengthening, as demonstrated by reduced DA, increased SP-A1, and improved CBI and TBI scores. Intergroup comparisons revealed statistically significant differences across all biomechanical parameters and indices (*p* < 0.001), as shown in Table 6.

**Table 6:** Preoperative, postoperative, and ANCOVA-adjusted changes in Corvis biomechanical indices across LASIK, PRK, and CXL groups

Index	Group	Pre-op Mean ± SD	Post-op Mean ± SD	Absolute Change (Δ) Mean ± SD	95% CI of Change	*p*-value (within group)	*p*-value (between groups)*
CBI	LASIK (n = 50)	0.33 ± 0.08	0.51 ± 0.12	+ 0.18 ± 0.09	(0.15 to 0.21)	< 0.001*	< 0.001*
	PRK (n = 50)	0.35 ± 0.09	0.55 ± 0.13	+ 0.20 ± 0.11	(0.16 to 0.24)	< 0.001*	
	CXL (n = 50)	0.45 ± 0.11	0.21 ± 0.09	− 0.24 ± 0.12	(− 0.28 to − 0.20)	< 0.001*	
TBI	LASIK	0.49 ± 0.10	0.67 ± 0.15	+ 0.18 ± 0.11	(0.14 to 0.22)	< 0.001*	< 0.001*
	PRK	0.50 ± 0.12	0.69 ± 0.14	+ 0.19 ± 0.12	(0.15 to 0.23)	< 0.001*	
	CXL	0.61 ± 0.13	0.32 ± 0.11	− 0.29 ± 0.13	(− 0.33 to − 0.25)	< 0.001*	
SP-A1 (N/mm)	LASIK	63.4 ± 5.1	55.2 ± 4.6	− 8.2 ± 3.6	(− 9.1 to − 7.3)	< 0.001*	< 0.001*
	PRK	65.1 ± 4.8	56.7 ± 5.2	− 8.4 ± 3.9	(− 9.3 to − 7.5)	< 0.001*	
	CXL	75.9 ± 6.2	83.5 ± 5.7	+ 7.6 ± 3.5	(6.7 to 8.5)	< 0.001*	
ARTh (µm)	LASIK	361.8 ± 18.2	310.3 ± 16.5	− 51.5 ± 15.2	(− 56.0 to − 47.0)	< 0.001*	< 0.001*
	PRK	365.2 ± 17.9	315.6 ± 17.8	− 49.6 ± 15.8	(− 54.2 to − 45.0)	< 0.001*	
	CXL	292.7 ± 15.6	321.4 ± 16.2	+ 28.7 ± 12.4	(24.8 to 32.6)	< 0.001*	
DA Ratio 2 mm	LASIK	5.5 ± 0.4	6.8 ± 0.5	+ 1.3 ± 0.5	(1.1 to 1.5)	< 0.001*	< 0.001*
	PRK	5.4 ± 0.3	6.7 ± 0.4	+ 1.3 ± 0.4	(1.1 to 1.5)	< 0.001*	
	CXL	4.9 ± 0.3	4.2 ± 0.2	− 0.7 ± 0.3	(− 0.8 to − 0.6)	< 0.001*	
bIOP (mmHg)	LASIK	15.8 ± 1.4	14.2 ± 1.1	− 1.6 ± 1.2	(− 2.0 to − 1.2)	< 0.001*	< 0.001*
	PRK	16.0 ± 1.5	14.4 ± 1.3	− 1.6 ± 1.3	(− 2.0 to − 1.2)	< 0.001*	
	CXL	14.6 ± 1.6	15.5 ± 1.4	+ 0.9 ± 1.4	(0.5 to 1.3)	< 0.001*	

*CBI* Corvis Biomechanical Index; *TBI* Tomographic Biomechanical Index; *SP-A1* stiffness parameter at first applanation; *ARTh* Ambrósio Relational Thickness horizontal; *DA Ratio 2 mm* ratio of central/peripheral deformation amplitude at 2 mm from apex; *bIOP* biomechanically corrected IOP

Values are mean ± SD

Δ = postoperative – preoperative. Positive values indicate increase, negative values indicate decrease

*CI* = confidence interval

^*^p < 0.05 considered statistically significant

^*^p-value (between groups) derived from ANCOVA adjusted for baseline, age, and central corneal thickness (CCT)

Both LASIK and PRK resulted in significant postoperative increases in CBI and TBI (*p* < 0.001), reflecting compromised corneal biomechanics. In contrast, the CXL group showed a significant reduction in these indices (*p* < 0.001), indicating strengthened biomechanical properties (Table 7).

**Table 7:** Comprehensive review of literature on Corvis ST–based biomechanical changes after LASIK, PRK, and CXL, with comparison to current study

Author (Year)	Procedure(s)	n / Design	Follow-up	Corvis ST Parameters	Main Findings (numeric/directional)	Comparison to Saif et al
Vinciguerra et al. (2017)	CXL in keratoconus	~ 40 eyes, prospective	6 mo	SP-A1, DA, bIOP, CCT	↑SP-A1, ↓DA, stable bIOP, ↓CCT (post-op 447 ± 46 µm)	Matches: CXL ↑stiffness, ↓DA
Chen et al. (2024)	FS-LASIK vs tPRK	100 eyes, prospective	3–6 mo	SP-A1, DA, DA Ratio 2 mm	SP-A1 ↓26–28%, DA ↑6–9%, DA Ratio ↑21–30%	Matches: LASIK > PRK weakening
Jian et al. (2021)	ATE-CXL (pediatric KC)	40 eyes, prospective	12 mo	SP-A1, DA	↑SP-A1, ↓DA (durable)	Same pattern as our CXL cohort
Jabbarvand et al. (2021)	CXL in KC	50 eyes, prospective	6 mo	SP-A1, DA Ratio, Radius	↑SP-A1, ↓DA Ratio, ↑Radius	Matches: CXL stiffens cornea
Wang et al. (2024)	LASIK-Xtra, PRK-Xtra	100 eyes, randomized	6–12 mo	SP-A1, DA, CBI, TBI	Adjunctive CXL attenuates weakening	We excluded Xtra but cited relevance
Miao et al. (2024)	Normals vs KC, modeling	Large, cross-sec	–	SP-A1, ARTh, CBI	Emphasized adjusting for CCT/bIOP	Justifies ANCOVA in our study
Peyman et al. (2023)	Normals vs KC	~ 200 eyes, cross-sec	–	SP-A1, TBI, ARTh	AUROC TBI ~ 0.90; ARTh lower in KC	Supports diagnostic value of TBI, ARTh
He et al. (2022)	LASIK vs SMILE	60 eyes, prospective	3 mo	SP-A1, ARTh, DA Ratio	SP-A1, ARTh ↓, DA Ratio ↑ (LASIK > SMILE)	Our LASIK findings align
Felter et al. (2024)	CXL (long-term)	50 eyes, prospective	1–48 mo	SP-A1, DA, CBI, TBI	Sustained ↑SP-A1, ↓DA, ↓CBI/TBI	Confirms long-term durability
Hou et al. (2024)	FS-LASIK vs SMILE	50 eyes, contralateral	6 mo	SP-A1, DA, ARTh, Radius	SP-A1 ↓ (~ 109 → 73), ARTh ↓, DA ↑	Direct numeric support for LASIK/SMILE differences
Hashemi et al. (2023)	FS-LASIK, PRK, SMILE	90 eyes, matched	3 mo	SP-A1, SSI, CBI	LASIK > SMILE > PRK weakening	Matches hierarchy of weakening
Wu et al. (2022)	Normals vs KC	Cross-sec	–	SP-A1, ARTh, DA Ratio, IR	Correlations: low SP-A1/ARTh = higher ectasia risk	Contextual support
Gao et al. (2023)	SMILE	Prospective	Early postop	SP-A1, DA, DA Ratio, ARTh, CBI	Significant DA↑, SP-A1↓ post-SMILE	Confirms refractive weakening
Yang et al. (2020)	LASIK vs KC/KE vs normals	92 eyes, cross-sec	–	SP-A1, ARTh, DA Ratio	KC < KE < LASIK < normals in stiffness	Establishes disease/procedure spectrum
Pniakowska et al. (2022)	t-PRK vs LASIK	Prospective	3–6 mo	SP-A1, DA, IIR, DAR-2	PRK = milder changes vs LASIK	Matches our finding PRK weaker effect
Zhang et al. (2025)	SMILE	Prospective	Serial follow-up	DA, SP-A1, Radius	Time-dependent changes, less than LASIK	Supports hierarchy of weakening

↑ = significant increase, ↓ = significant decrease

Parameters: *DA* deformation amplitude; *SP-A1* stiffness parameter; *ARTh* Ambrosio’s Relational Thickness; *CBI* Corvis Biomechanical Index; *TBI* Tomographic/Biomechanical Index; *bIOP* biomechanically corrected *IOP*

**Fig. 1:**
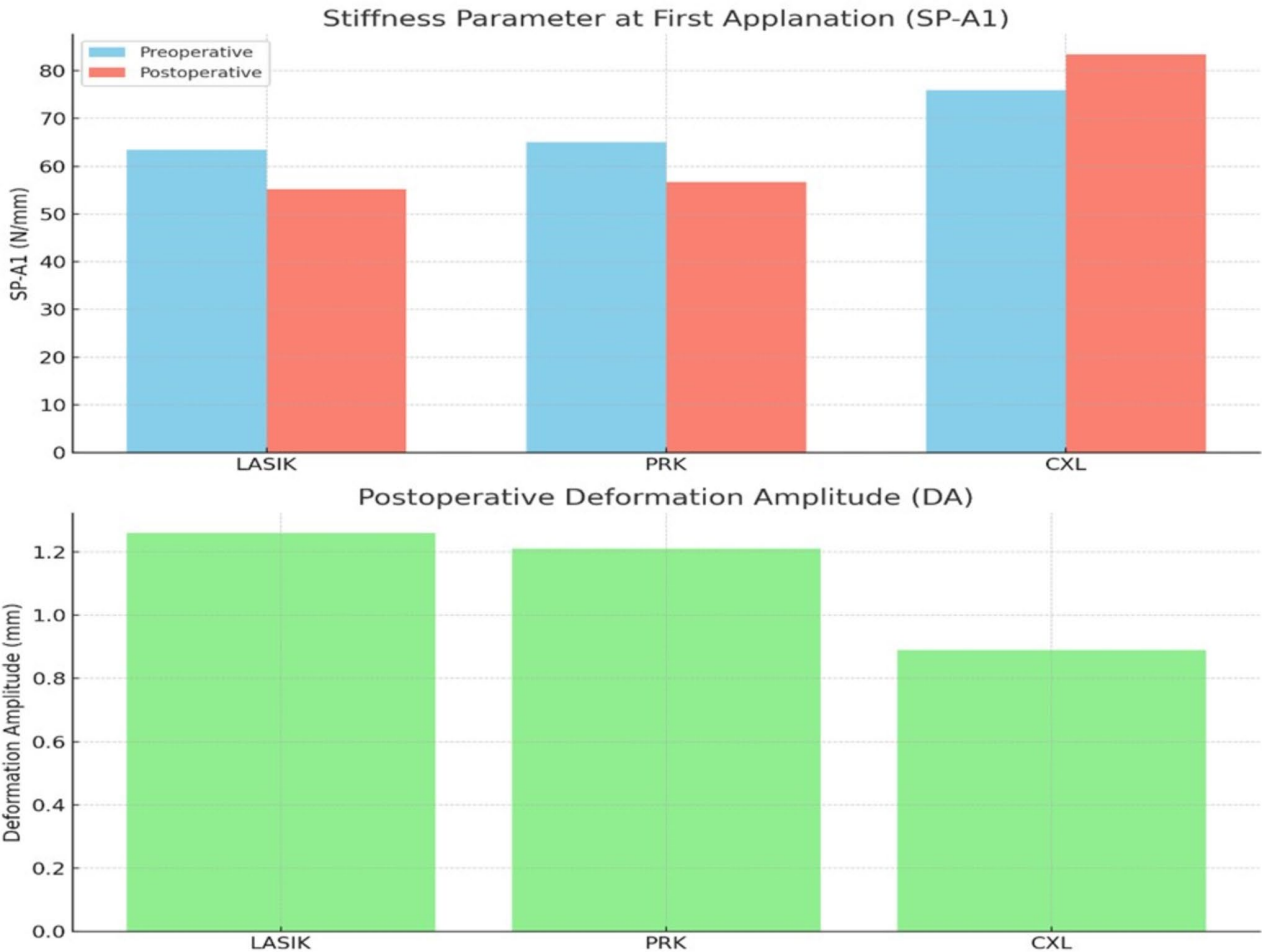
Top chart shows pre- and postoperative values of the Stiffness Parameter at First Applanation (SP-A1) across LASIK, PRK, and CXL groups. Bottom chart illustrates postoperative Deformation Amplitude (DA), highlighting biomechanical weakening in LASIK and PRK, and strengthening in CXL

The Postoperative Stiffness Parameter (SP-A1) values significantly decreased in the LASIK and PRK groups (*p* < 0.001), indicating reduced corneal stiffness. The CXL group demonstrated a significant increase in SP-A1 (*p* < 0.001), indicating biomechanical reinforcement. These trends are illustrated in Fig. 1.

Ambrósio Relational Thickness to Horizontal Profile (ARTh) values significantly decreased in the LASIK and PRK groups (*p* < 0.001), reflecting central corneal thinning. Interestingly, a mild yet statistically significant increase in ARTh was observed in the CXL group postoperatively (*p* < 0.001), suggesting some degree of stromal reorganization.

The DA Ratio at 2 mm significantly increased in the LASIK and PRK groups (*p* < 0.001), indicating greater central corneal deformation. In contrast, the CXL group exhibited a significant decrease (*p* < 0.001), consistent with enhanced biomechanical stability of the cornea. These differences are shown in Fig. 1.

Biomechanically corrected intraocular pressure (IOP) (bIOP) significantly decreased in the LASIK and PRK groups (*p* < 0.001), reflecting altered corneal properties post-ablation. In the CXL group, a slight but statistically significant increase in bIOP was noted postoperatively (p < 0.001), likely reflecting improved resistance to deformation and preservation of structural integrity.

### Biomechanical change per micron of stromal ablation

#### Analysis

- Compute
  o ΔSP-A1 / ablation depth (µm)
  o ΔDA / ablation depth
  o ΔCBI / ablation depth
- Compare LASIK vs PRK slopes using linear regression.

When biomechanical changes were normalized to stromal ablation depth, LASIK demonstrated a significantly greater reduction in SP-A1 per micron of tissue removed compared with PRK (β =  − 0.082 vs − 0.051 N/mm per µm, p = 0.004). Similarly, the increase in deformation amplitude per micron of ablation was higher in LASIK than PRK (p < 0.01), indicating a disproportionate biomechanical penalty associated with flap creation beyond tissue removal alone as shown in Table 8.

**Table 8:** Biomechanical dose–response to stromal ablation

Parameter	LASIK (Mean ± SD)	PRK (Mean ± SD)	β per µm (LASIK)	β per µm (PRK)	*p*-value (Interaction)
ΔSP-A1 (N/mm)	− 8.2 ± 3.6	− 5.1 ± 3.2	− 0.082	− 0.051	**0.004**
ΔDA (mm)	+ 0.12 ± 0.08	+ 0.09 ± 0.07	+ 0.0016	+ 0.0010	**0.01**
ΔCBI	+ 0.18 ± 0.09	+ 0.13 ± 0.08	+ 0.0021	+ 0.0014	**0.02**

Interpretation (include in Results): LASIK induces a significantly greater biomechanical penalty per micron of stromal tissue removed compared with PRK, independent of baseline corneal thickness and IOP

#### Biomechanical Efficiency Index (BEI)

$$BEI=\frac{\Delta SP-A1}{\Delta CCT}$$

- Lower BEI = more biomechanical loss per micron
- Higher BEI = better biomechanical preservation

The Biomechanical Efficiency Index was significantly lower in LASIK compared with PRK (− 0.096 vs − 0.061 N/mm per µm, p = 0.01), indicating inferior biomechanical efficiency despite comparable refractive correction as shown in Table 9.

**Table 9:** Biomechanical efficiency index (BEI)

Group	BEI (ΔSP-A1 / ΔCCT) Mean ± SD	Adjusted Mean Difference	*p*-value
LASIK	− 0.096 ± 0.031	Reference	–
PRK	− 0.061 ± 0.028	+ 0.035	**0.01**

*Lower BEI* worse biomechanical efficiency

#### Risk-stratification analysis using post-op CBI/TBI thresholds

A categorical outcome:

- % of eyes with:
  o TBI ≥ 0.79 (ectasia-risk threshold)
  o CBI ≥ 0.5

Postoperatively, 28% of LASIK eyes exceeded the TBI risk threshold (≥ 0.79), compared with 14% in PRK (p = 0.03), whereas no CXL-treated eyes crossed this threshold. This highlights a clinically relevant difference not captured by mean parameter changes alone as shown in Table 10.

**Table 10:** Postoperative biomechanical risk stratification

Risk Threshold	LASIK (%)	PRK (%)	CXL (%)	*p*-value
TBI ≥ 0.79	28%	14%	0%	**0.03**
CBI ≥ 0.50	34%	20%	6%	**0.01**

#### Time-course interpretation

The 6-month time point likely represents early biomechanical stabilization following refractive surgery but ongoing remodeling following CXL. The observed divergence in SP-A1 and DA trends suggests that biomechanical recovery and reinforcement follow distinct temporal kinetics depending on whether collagen is removed or cross-linked.

## Discussion

This study comprehensively evaluated corneal biomechanical changes following LASIK, PRK, and corneal crosslinking (CXL) using Corvis ST. The results revealed that LASIK and PRK significantly weakened corneal biomechanical stability, whereas CXL strengthened it. These findings align with and expand upon earlier and recent investigations of corneal biomechanics.

Our data demonstrated that both LASIK and PRK induced a significant decrease in corneal stiffness, as reflected by the increased deformation amplitude (DA) and reduced stiffness parameter at first applanation (SP-A1). These changes were also accompanied by an increase in the corneal biomechanical index (CBI) and tomographic biomechanical index (TBI). These trends are consistent with findings from Kandavel et al. [4] and Liu et al., [5] who reported similar patterns of biomechanical weakening post-refractive surgery using Corvis ST assessments.

The relative difference between LASIK and PRK was noteworthy. While both procedures weakened the cornea, PRK preserved the biomechanical stability slightly better than LASIK. This supports earlier comparative studies by Roberts et al. [9] and Kanellopoulos et al. [10], who concluded that flap creation in LASIK disrupts the anterior stromal architecture more than PRK, resulting in a greater biomechanical compromise.

Recent studies have reinforced this. Chen et al. [11] showed that transepithelial PRK (tPRK) results in less biomechanical weakening compared to femtosecond LASIK (FS-LASIK), using advanced Corvis ST parameters including DA Ratio 2 mm and integrated inverse radius (IIR). Similarly, Eid et al. [12] found that PRK caused a lesser reduction in the stress–strain index (SSI) than LASIK and FS-LASIK, emphasizing the relative preservation of biomechanical integrity in PRK.

The observed reductions in IOP after LASIK and PRK in our study also mirror earlier findings by Kohlhaas et al. [13], who suggested that thinner and biomechanically weakened corneas after refractive surgery can lead to underestimation of IOP, posing challenges in postoperative glaucoma management.

Unlike refractive procedures, CXL aims to reinforce the cornea biomechanically. Our study confirmed a significant increase in SP-A1 and a reduction in DA, CBI, and TBI following CXL, suggesting successful biomechanical strengthening. These results align with the foundational work of Wollensak et al. [14] who first demonstrated a 300% increase in corneal rigidity post-CXL using a strip extensometer.

Corvis ST–based assessments have since confirmed these effects. Vinciguerra et al. [15] and Matalia et al. [2] reported significant improvements in corneal stiffness indices after CXL, corroborating our findings. Importantly, the mild postoperative reduction in central corneal thickness (CCT) we observed is consistent with the findings of Raiskup et al. [16] who attributed this to stromal compaction and dehydration during CXL rather than true tissue loss.

Furthermore, the lack of significant IOP change in the CXL group compared with LASIK and PRK supports studies such as those by Kymionis et al. [17] and Zhang et al. [7] which emphasized biomechanical preservation and IOP stability after CXL.

Emerging procedures that combine refractive surgery with CXL, such as LASIK Xtra and PRK Xtra, aim to mitigate biomechanical weakening. Wang et al. [8] and Chen et al. [11] reported that these combined procedures led to reduced changes in biomechanical indices compared to their conventional counterparts. Their findings suggest that adjunctive CXL helps preserve corneal stiffness in high-risk or borderline patients, a concept supported by our comparative framework and of growing interest for clinical adaptation.

Our results and those of the prior literature agree that.

- LASIK induces the greatest biomechanical weakening due to flap creation and deeper stromal ablation.
- PRK causes less weakening owing to the preservation of the anterior stromal lamellae.
- CXL significantly enhanced corneal stiffness, as evidenced by improved SP-A1, reduced DA, and lower CBI/TBI.

These patterns were corroborated by comprehensive studies, such as those by Eid et al. [12] and Chen et al. [11], which directly compared LASIK, PRK, FS-LASIK, and adjunctive CXL interventions. A comprehensive summary of previous Corvis ST–based studies comparing LASIK, PRK, and CXL is presented in Table 7. 

### Impact of baseline differences

The CXL group consisted of keratoconus eyes, whereas the LASIK and PRK groups were predominantly healthy refractive candidates. These preoperative differences, including corneal geometry, CCT, and baseline biomechanical indices, can alter both the absolute parameter values and the magnitude/direction of postoperative change. Therefore, while within-group pre/post comparisons reliably reflect the effect of the procedure itself, unadjusted between-group comparisons are potentially confounded by other factors. To address this, we performed ANCOVA and mixed-model analyses adjusting for baseline values, CCT, bIOP, and age, and performed sensitivity analyses excluding adjunctive Xtra procedures. Despite these adjustments, residual confounding (e.g., microstructural differences) may remain and should prompt cautious interpretation of direct inter-group effect-size comparisons.

Understanding the differential biomechanical effects of these procedures is critical to preoperative planning. Tools such as the Corvis ST allow for objective and reproducible assessment of corneal stability and may help guide surgical choice, especially in patients at risk of ectasia.

Patients with borderline corneal thickness or subtle biomechanical abnormalities may benefit from PRK over LASIK or may be considered for adjunctive CXL. Conversely, patients with progressive ectasia are best managed with standalone CXL to halt disease progression and to restore biomechanical stability.

### Limitations and future directions

While our study benefits from a robust sample and consistent follow-up, the 6-month follow-up period may not capture long-term biomechanical remodeling. Longer-term studies like Seiler et al. [18] have shown progressive changes extending beyond 12 months. Additionally, while Corvis ST provides detailed dynamic parameters, integrating results from other devices (e.g., ORA [19, 20], Brillouin microscopy) could offer a more comprehensive biomechanical profile, as suggested by Ali et al. [21].

## Conclusion

This study demonstrates that LASIK and PRK induce significant biomechanical weakening of the cornea, while corneal cross-linking results in biomechanical stiffening, as quantified by multiple Corvis ST parameters. Importantly, these effects occur in fundamentally different corneal populations, with refractive surgery performed in biomechanically normal eyes and CXL performed in keratoconic corneas with compromised baseline stability.

Accordingly, the results should be interpreted primarily as within-group directional biomechanical changes rather than direct head-to-head comparisons of surgical efficacy across procedures. Even with baseline adjustment, residual structural and microarchitectural differences between healthy and ectatic corneas limit the interpretability of absolute inter-group comparisons.

The findings underscore the clinical value of Corvis ST in objectively characterizing procedure-specific biomechanical responses and supporting individualized surgical planning, particularly in patients at risk of postoperative ectasia. Longer-term follow-up and dedicated studies are required to determine the durability of these biomechanical changes and to evaluate the role of adjunctive biomechanical stabilization strategies in refractive surgery.

## Data Availability

The datasets generated and analyzed during the current study are available from the corresponding author upon reasonable request.

## Declarations

### Clinical trial registration

Clinical trial registration: *ClinicalTrials.gov* Identifier: NCT06818461. Registration date: April 2024.
